# Reply: Matters Arising ‘Lewy body disease or diseases with Lewy bodies?’

**DOI:** 10.1038/s41531-022-00338-3

**Published:** 2022-06-23

**Authors:** Kateřina Menšíková, Radoslav Matěj, Carlo Colosimo, Raymond Rosales, Lucie Tučková, Jiří Ehrmann, Dominik Hraboš, Kristýna Kolaříková, Radek Vodička, Radek Vrtěl, Martin Procházka, Martin Nevrlý, Michaela Kaiserová, Sandra Kurčová, Pavel Otruba, Petr Kaňovský

**Affiliations:** 1 Department of Neurology, University Hospital, Palacky University, Olomouc, Czech Republic; 2 Department of Neurology, Faculty of Medicine and Dentistry, Palacky University, Olomouc, Czech Republic; 3 Department of Pathology and Molecular Medicine, 3rd Faculty of Medicine, Charles University, Prague, Czech Republic; 4 Department of Neurology, Santa Maria University Hospital, Terni, Italy; 5 The Neuroscience Institute, Department of Neurology and Psychiatry, University of Santo Tomás Hospital, Manila, Philippines; 6 Department of Clinical and Molecular Pathology, Faculty of Medicine and Dentistry, Palacky University, Olomouc, Czech Republic; 7 Department of Clinical Genetics, University Hospital, Palacky University, Olomouc, Czech Republic

**Keywords:** Parkinson's disease, Parkinson's disease

**REPLYING TO** K. A. Jellinger. *npj Parkinsons Disease* 10.1038/s41531-022-00337-4 (2022)

We have read with interest the “Matters Arising” text of Kurt Jellinger, in which he comments on some parts of our recent paper^1^. To our statements: “a) that from the strict pathological point of view, there is practically no difference between PD and PDD; even the experienced neuropathologist is not able to differentiate, and (b) that there is no sharp pathological border between PDD and DLB, although the degree of AD pathology and the presence of CAA are probably the most significant pathological differences between these two phenotypes“ adds Dr. Jellinger exceptionally valuable personal experience gained from decades of neuropathological research in parkinsonism and atypical parkinsonism. We are aware of his seminal works from 2007 and 2010 in which he reported autopsy findings in parkinsonian patients and also the differences between findings in those demented and those not^2,3^. Indeed, we have also (very recently) carefully studied his last paper on this topic from 2022^4^, in which he has broadly discussed the study of Hansen et al.^5^.

We believe that our present work corroborates with the Dr. Jellinger’s findings and related inferences from the point of view of neuropathology of parkinsonism. The issues we raised were in the regard to reliability of current clinical diagnostic criteria of the LBD phenotypes, which we believe are either outdated or – at best – speculative. Of course, there might be a broad spectrum of cognitive deterioration present in parkinsonism, from mild cognitive impairment to severe dementia. This spectrum seems to be pathologically reflected in the given degree of Braak’s LBD staging, however, it is apparently not reflected in the beta-amyloid load.

Moreover, except of β-amyloid load and CAA pathology, there is an increasing evidence that more different co-pathologies in either LBD or PD as well as other neurodegenerative disorders can be considered^6^. From the clinical point of view, this fact substantially compromises the concept of disease-specific phenotype and makes clinical diagnosis more difficult^7–9^. This is already known from the daily practice, nevertheless, it should be confirmed in much larger cross-correlation studies.

We therefore welcome the debated issues raised by Dr. Jellinger regarding our concept of the existence of only two “Lewy body diseases“, Parkinson’s disease and dementia with Lewy bodies; the mutual relationships of Lewy pathology and β-amyloid and CAA pathology in both these phenotypes were excellently described in his comment too.
